# Consequences at adulthood of transient inactivation of the parahippocampal and prefrontal regions during early development: new insights from a disconnection animal model for schizophrenia

**DOI:** 10.3389/fnbeh.2014.00118

**Published:** 2014-04-07

**Authors:** Francisca Meyer, Alain Louilot

**Affiliations:** ^1^Department of Molecular Animal Physiology, Donders Institute for Brain, Cognition and Behaviour, Radboud University NijmegenNijmegen, Netherlands; ^2^INSERM U 1114, Faculty of Medicine, FMTS, University of StrasbourgStrasbourg, France

**Keywords:** schizophrenia, animal modeling, neonatal functional inactivation, entorhinal cortex, ventral subiculum, prefrontal cortex, latent inhibition

## Abstract

The psychic disintegration characteristic of schizophrenia is thought to result from a defective connectivity, of neurodevelopmental origin, between several integrative brain regions. The parahippocampal region and the prefrontal cortex are described as the main regions affected in schizophrenia. Interestingly, latent inhibition (LI) has been found to be reduced in patients with schizophrenia, and the existence of a dopaminergic dysfunction is also generally well accepted in this disorder. In the present review, we have integrated behavioral and neurochemical data obtained in a LI protocol involving adult rats subjected to neonatal functional inactivation of the entorhinal cortex, the ventral subiculum or the prefrontal cortex. The data discussed suggest a subtle and transient functional blockade during early development of the aforementioned brain regions is sufficient to induce schizophrenia-related behavioral and dopaminergic abnormalities in adulthood. In summary, these results support the view that our conceptual and methodological approach, based on functional disconnections, is valid for modeling some aspects of the pathophysiology of schizophrenia from a neurodevelopmental perspective.

## Introduction

### Schizophrenia

Schizophrenia is a complex neuropsychiatric disorder of unknown etiology, and its pathophysiology remains poorly understood. It affects about 1% of the population worldwide (Insel, 2010) and is a heavy burden not only for the families of the patients but also for society (Knapp et al., 2004; McEvoy, 2007). It was recently proposed that the characteristic psychic disintegration observed in schizophrenia would result from abnormal connectivity, i.e., disconnections, at least partly of neurodevelopmental origin (Weinberger and Lipska, 1995; Andreasen, 1999; Lewis and Levitt, 2002; Sawa and Snyder, 2002; Insel, 2010; Rapoport et al., 2012) between different integrative brain regions, especially the prefrontal cortex, the medial temporal lobe and striatal regions (Lawrie et al., 2002; Stephan et al., 2009; Meyer-Lindenberg, 2010; Fornito et al., 2011; Schmitt et al., 2011). This “disconnection hypothesis” in schizophrenia is backed up by a great many of neurophysiological and neuroimaging studies, but the cause of the dysconnectivity is still the subject of discussion. The current debate centers on two possible explanations, namely that the dysconnectivity may result from either (1) abnormal synaptic plasticity; or (2) abnormal white matter connections between two integrative brain regions (Friston, 1998, 1999; Stephan et al., 2006, 2009; for review see Shi et al., 2012). Both abnormal dendritic spine density (Glantz and Lewis, 2000; Black et al., 2004; Kolluri et al., 2005), and also myelination abnormalities (Du et al., 2013; Palaniyappan et al., 2013) have been reported in patients with schizophrenia. Early developmental abnormalities could lead to dopamine dysregulation (Murray et al., 2008; O’Donnell, 2011; Eyles et al., 2012) which ultimately results in the well-acknowledged dopaminergic imbalance observed in patients with schizophrenia (Meltzer and Stahl, 1976; Carlsson et al., 2001; Kuepper et al., 2012). Importantly, the prefrontal cortex and medial temporal lobe (i.e., entorhinal cortex, ventral subiculum) stand out as the main regions affected in schizophrenia. In this respect several cytoarchitectural and neuronal morphometric abnormalities have been described at the level of the prefrontal cortex (Garey, 2010; Yang et al., 2011; Nesvåg et al., 2012; Palaniyappan and Liddle, 2012), the entorhinal cortex (Arnold, 2000; Falkai et al., 2000; Prasad et al., 2004) and the ventral subiculum (Arnold, 2000; Rosoklija et al., 2000; Law et al., 2004).

### Neurodevelopmental animal models for schizophrenia: focus on neonatal disconnection animal models

An accumulation of evidence over the past 20 years in favor of the neurodevelopmental hypothesis for schizophrenia has resulted in a number of animal models based on early impairment of brain development (for reviews see Weinberger, 1996; Rehn and Rees, 2005; Powell, 2010). It is not possible to present them all in this article, but, basically, these neurodevelopmental animal models for schizophrenia can be divided up into (i) epidemiological models; (ii) genetic models; and (iii) heuristic models. Epidemiological animal models are derived from studies which point to an increased risk of developing schizophrenia following perinatal exposure to environmental insults (e.g., isolation rearing, maternal deprivation, or postnatal exposure to stress, e.g., Ellenbroek et al., 2004; Lee et al., 2007; Amitai et al., 2013), infection or immune activation (Fruntes and Limosin, 2008; Meyer, 2014), nutritional deficiencies (Palmer et al., 2004; Harms et al., 2008), as well as obstetric complications (Boksa, 2004; Juarez et al., 2008). Recently developed genetic models are based on findings that implicate developmental candidate genes (e.g., Reelin, STOP, DISC1, NRG1) or human copy number variations (CNVs; e.g., 22q11.2 deletion; Chen et al., 2006; Powell et al., 2009; Fénelon et al., 2013), whereas heuristic models take account of a wider array of clinical and biological findings relevant to the pathophysiology of schizophrenia. Some of these heuristic models aim to reproduce cytoarchitectural abnormalities observed in schizophrenia and thus involve impaired neurogenesis based on the use of antimitotic agents such as methylazoxymethanol acetate (MAM; see Lodge and Grace, 2009) or cytosine arabinoside (Ara-C; Elmer et al., 2004) administered at the end of the gestational period, and more specifically at gestational day (GD) 17 or GD 19.5–20.5 respectively, a period corresponding to the end of the first trimester in humans (Clancy et al., 2001). One of the best-characterized neurodevelopmental animal models for schizophrenia is the neonatal ventral hippocampal lesion model (NVHL), which was initially developed to take account of anatomopathological data observed in the hippocampus of patients with schizophrenia (Lipska et al., 1993; Lipska and Weinberger, 1994; Lipska, 2004; Tseng et al., 2009). In this model, an excitotoxic lesion of the ventral hippocampus was performed at postnatal day 7 (PND7), which corresponds to the middle of the second trimester of gestation in humans (Clancy et al., 2001), considered to be a period of high vulnerability for developing schizophrenia (Weinberger and Lipska, 1995; Lewis and Levitt, 2002; Tseng et al., 2009). However, although the heuristic validity of this animal model is undeniable, it relies on massive and irreversible damage of the ventral hippocampus (Lipska et al., 1993; Fatemi and Folsom, 2009), whereas post-mortem brain analyzes performed on schizophrenia patients have revealed only subtle anatomical alterations in the hippocampus, but no lesions or tracks of lesions (Harrison, 1999, 2004). Other neurodevelopmental models have been devised involving a neonatal excitotoxic lesion of another temporal region, the entorhinal cortex (Harich et al., 2008) or medial prefrontal cortex (Bennay et al., 2004; Schwabe et al., 2004, 2006; Enkel and Koch, 2009). However, as with the ventral hippocampus no lesions or tracks of lesions have been observed in these regions in patients with schizophrenia (Harrison, 1999). To overcome the construct validity weakness of lesion-based approaches (Lipska and Weinberger, 2000; Tseng et al., 2009), postnatal transient functional inactivation models have been designed as alternative models based on reversible neonatal functional blockade induced by local intracerebral infusion of tetrodotoxin (TTX), a well-known blocker of voltage-sensitive sodium channels (Stevens et al., 2011). Electrical activity appears to be essential during neonatal brain development (Spitzer, 2006), and an interruption of impulse activity by TTX has been reported to have inhibitory effects on myelination (Demerens et al., 1996) and to disrupt the refinement of synaptic connections in target structures (Stryker and Harris, 1986; Katz and Shatz, 1996), as well as the normal maturing of dendritic spines (Drakew et al., 1999; Frotscher et al., 2000). Thus, the consequences of neonatal TTX inactivation appear to be adequate for modeling disconnections as proposed for schizophrenia (see above). Moreover, it has been shown that neonatal transient inactivation of the ventral hippocampus leads to schizophrenia-relevant features in adulthood, such as motor hyperactivity following a pharmacological (D-amphetamine, MK-801) or environmental challenge (exposure to a novel environment; Lipska et al., 2002). Interestingly, in adult rats that underwent neonatal TTX inactivation of the ventral hippocampus, Brooks and co-workers reported deficient acetylcholine release from the prefrontal cortex following mesolimbic stimulation (Brooks et al., 2011), as well as deficits in a set-shifting task (Brooks et al., 2012). In addition, results obtained in our laboratory, which are discussed in detail below, showed that neonatal transient TTX inactivation of the entorhinal cortex, ventral subiculum or prefrontal cortex induced disturbed dopaminergic and behavioral responses related to latent inhibition (LI) in adulthood (Peterschmitt et al., 2007; Meyer et al., 2009; Meyer and Louilot, 2011, 2012). It is important to note that no lesions or macroscopic anatomical changes have been observed in the aforementioned studies following neonatal TTX inactivation (Lipska et al., 2002; Peterschmitt et al., 2007; Meyer et al., 2009; Brooks et al., 2011; Meyer and Louilot, 2011, 2012; Brooks et al., 2012; Usun et al., 2013). Taken together, functional disconnection models appear to be a relevant conceptual approach to animal modeling for some aspects of the pathophysiology of schizophrenia and without inducing any major anatomical lesion.

### Latent inhibition

Some data point towards disturbed information processing in patients with schizophrenia. In this respect, the behavioral paradigms most widely used in schizophrenia research are LI and prepulse-inhibition of the startle reflex (PPI). Although both LI and PPI have been reported to be disrupted in acute schizophrenia patients (Baruch et al., 1988; Gray et al., 1995; Braff et al., 1999, 2001; Rascle et al., 2001; Kumari and Ettinger, 2010), whereas a contradictory view exists (Swerdlow, 2010), PPI is also impaired in a variety of other disorders such as Huntington’s disease, Tourette’s syndrome, temporal lobe epilepsy with psychosis, and post-traumatic stress disorder (Braff et al., 2001). By contrast, LI disruption appears more specific to schizophrenia (see Lubow and Weiner, 2010) and is a cognitive marker of choice for the animal modeling of schizophrenia. As first described by Lubow and Moore (Lubow and Moore, 1959), LI is a behavioral phenomenon observed in several animal species including humans (Lubow, 1989). It was originally defined as retarded acquisition of the conditioned response (CR) when the conditional stimulus (CS) is first pre-exposed on its own. It is generally accepted that LI allows for adaptations to be made to a changing environment. Despite the relative simplicity of the LI phenomenon, theoretical explanations have proved difficult. Since its discovery, different theories (see Lubow and Weiner, 2010) have been proposed, explaining it as (1) a defect in the acquisition of conditioning (*attention/associability theories*); (2) a switching mechanism—controlled by the hippocampal formation—between the CS-reinforcement associations acquired during conditioning and the CS-no event associations acquired during pre-exposure (*Switching model*); or (3) a defect in the expression of conditioning (*retrieval theories*). As for the neurobiological substrates of LI, lesion studies and *in vivo* neurochemical approaches (i.e., *in vivo* microdialysis, *in vivo* voltammetry) revealed the involvement of the mesencephalic dopaminergic systems (for review see Louilot et al., 2010) and in particular the dopaminergic neurons innervating the *core* and *dorsomedian shell* part of the nucleus accumbens—with the *ventromedial* *shell* involved in the affective perception of the stimulus (Jeanblanc et al., 2002)—as well as the anterior part of the *dorsal striatum* (Jeanblanc et al., 2003).

## Disrupted latent inhibition: a recognition memory deficit?

**Figure 1:**
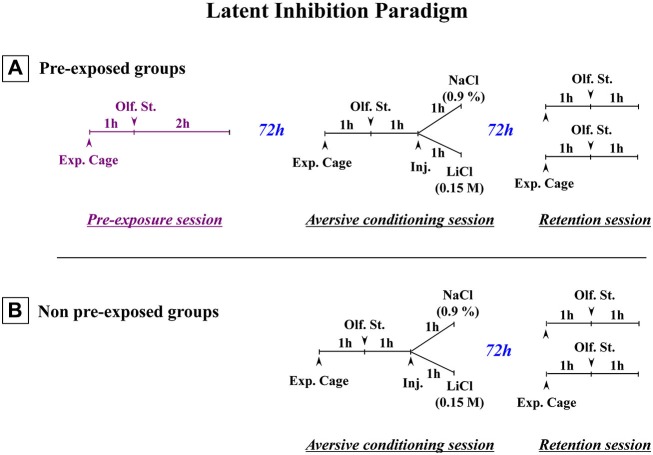
**Schematic representation of the paradigm used to obtain latent inhibition. (A)** Three-stage latent inhibition paradigm. During the pre-exposure (first) session, the animals were placed in the experimental cage (Exp. Cage) for 1 h without any olfactory stimulus (Olf. St.), before being exposed to the to-be-conditioned olfactory stimulus (banana odor) for 2 h. 72 h later, the animals were subjected to the conditioning (second) session. After a 1-h period that allowed the rats to become accustomed to the experimental cage, they were then exposed to the banana odor (CS) for 1 h. After that, they were given an intraperitoneal (i.p.) injection of either a saline (NaCl 0.9%) or an isotonic solution of LiCl (0.15 M). They then remained in the cage with the olfactory stimulus for a further hour. 72 h later, they were returned to the experimental cage for the test (third) session. After a 1-h familiarization period, they were exposed to the CS for a further hour. Their attraction or aversion towards the olfactory stimulus (banana odor) was then assessed in terms of how much time they spent near the olfactive source. The olfactory aversive conditioning protocol **(B)** was exactly the same except that non-pre-exposed animals were subjected only to the conditioning (second) session and the test (third) session (Adapted from Jeanblanc et al., 2002).

The present review includes behavioral and neurochemical data recently obtained in a LI protocol involving adult rats subjected to early neonatal (postnatal day 8) functional TTX inactivation of the entorhinal cortex, ventral subiculum or prefrontal cortex (Peterschmitt et al., 2007; Meyer et al., 2009; Meyer and Louilot, 2011, 2012). We were able to show that subtle and transient functional inactivation of the aforementioned cerebral regions during early development is sufficient to induce schizophrenia-related behavioral abnormalities: disrupted LI accompanied by dopaminergic changes recorded during adulthood in the dorsal striatum and the core part of the nucleus accumbens (see summarizing Table 1). In the context of these studies, LI was measured in a three-stage paradigm involving a conditioned olfactory aversive procedure with banana odor as the conditional stimulus (CS) and lithium chloride (LiCl), a nausea-induced toxic substance, as the unconditional stimulus (US; Jeanblanc et al., 2002; see Figure 1). This paradigm clearly allows for the observation of LI, as evidenced by the disappearance of the aversively conditioned behavioral response (i.e., aversion towards the CS) in the pre-exposed conditioned animals (see Jeanblanc et al., 2002; Peterschmitt et al., 2007; Louilot et al., 2010; Meyer and Louilot, 2011, 2012).

**Table 1:** **Summary of the main results obtained at the behavioral and neurochemical level following neonatal TTX inactivation of the entorhinal cortex, the ventral subiculum or the prefrontal cortex**.

**Brain regions inactivated at PND8**	**Reversal of behavioral LI expression**	**Reversal of LI-related dopaminergic responses**		**References**
		**Core**	**Dorsal striatum**	
Entorhinal cortex	√	√	Partial	Peterschmitt et al., 2007; Meyer et al., 2009
Ventral subiculum	√	Partial	√	Meyer et al., 2009; Meyer and Louilot, 2011
Prefrontal cortex	√	√	Partial	Meyer and Louilot, 2012; Meyer and Louilot, unpublished data

Interestingly, as regards the entorhinal cortex and ventral subiculum, the behavioral responses obtained following TTX inactivation of the two temporal regions performed at postnatal day 8 were similar or even stronger than those obtained following TTX inactivation of the same brain regions in adulthood prior to the pre-exposure session. Previous data obtained in adult animals suggested that both temporal regions are part of a system involved in the recognition memory of the stimulus (Jeanblanc et al., 2004; Peterschmitt et al., 2005, 2008). In this context, it is important to recall that in the past different theoretical constructs have been proposed as explanations for LI (see Lubow and Weiner, 2010). In short, it was initially suggested that LI reflects a delay in acquiring the conditioning relating to a learned inattention to the CS (see Lubow, 1989) or a decrease in CS associability (Mackintosh, 1975, 1983; Wagner, 1976) due to presentation of the CS alone during the pre-exposure session. Contrary to that, however, it has also been suggested that LI corresponds to a defect in the behavioral expression of conditioning which is normally acquired during the conditioning session (Miller and Matzel, 1988; Weiner, 1990, 2003; Kraemer et al., 1991; Bouton, 1993). Hypothesizing a failure in the expression of conditioning is an easier interpretation of the results obtained with our three-stage LI paradigm (see Figure 1) than hypothesizing a defect in the acquisition of conditioning. First of all, these results, which show that in the retention session pre-exposed conditioned animals display a similar approach to the CS (banana odor) as pre-exposed control and non-pre-exposed control animals (Jeanblanc et al., 2002), suggest a similar interest in the CS, for the three groups and therefore do not support the hypothesis of a learned inattention to the stimulus in the pre-exposed conditioned group. Secondly, results obtained previously in the different subregions of the nucleus accumbens (Jeanblanc et al., 2002) showed that dopaminergic responses in the core and dorsomedial shell parts of the nucleus accumbens are related to LI, whereas dopaminergic responses in the ventromedial shell part of the nucleus accumbens appear to be related only to conditioning, not LI, insofar as similar small dopaminergic increases were observed in this subregion in pre-exposed conditioned and non-pre-exposed conditioned groups whereas marked dopaminergic increases were observed in the two control groups. These latter results suggest aversive conditioning was normally acquired in pre-exposed conditionned animals but not behaviorally expressed during the retention (test) stage. This proposal is consistent with theoretical constructs which suggest differently that the expression of LI reflects interference or competition between the memories of the CS-alone or the CS-no event acquired during pre-exposure and the memory of the aversive valence related to the CS acquired during conditioning (Miller and Matzel, 1988; Bouton, 1993; see also Weiner, 2003; Louilot et al., 2010). In keeping with these explanations, in a heuristic perspective, one interpretation of the fact that conditioning is normally acquired but not expressed during the retention session would be that LI responses, at least in our LI paradigm, reflect the inhibitory influence a neuronal system involved in some kind of recognition memory of the CS alone or the CS-no event encoded during the pre-exposure session has upon a second neuronal system involved in the expression of aversive conditioning towards the CS (Jeanblanc et al., 2002). Past data suggest the basolateral nucleus of amygdala may be part of the second system (Louilot and Besson, 2000). To shore up our heuristic hypothesis regarding the system involved in recognition memory, we investigated in adult animals the consequences of functional inactivation of the entorhinal cortex and ventral subiculum, two structures described as being involved in both LI (Gray et al., 1995; Weiner and Feldon, 1997; Weiner, 2003) and recognition memory of olfactory stimuli (Suzuki and Eichenbaum, 2000; Petrulis et al., 2005; Eichenbaum et al., 2007). In so doing, we considered that if a structure is involved in encoding information during pre-exposure, it may also be involved in retrieving this information during the retention session. Thus, TTX inactivation of the entorhinal cortex and ventral subiculum was performed during the pre-exposure session, and the behavioral and dopaminergic responses were measured during the retention (third) session (Jeanblanc et al., 2004; Peterschmitt et al., 2005, 2008). A reversal of LI responses towards conditioned aversive responses was observed during this last session, lending support to the proposal that a neural system involved in recognition memory played a role in our LI paradigm. It is a proposal backed up by data obtained in a three-stage cued fear conditioning LI paradigm in adult mice following functional inactivation of the entorhinal/subicular region by muscimol (Lewis and Gould, 2007). More specifically, these authors showed that inactivation during the pre-exposure or retention session, but not conditioning session, causes the behavioral expression of LI to be lost during testing (see Peterschmitt et al., 2008, for an extensive discussion). Others have also suggested some kind of recognition memory is involved in LI, based on their findings in an inhibitory avoidance paradigm after cholinergic manipulation of the insular cortex at pre-exposure (Miranda and Bermúdez-Rattoni, 2007). In other words, there is support for the view that defective information retrieval related to the CS is involved in the disruption of LI. It is important to note here that (1) recognition memory defects have been reported in patients with schizophrenia (Danion et al., 1999; Huron and Danion, 2002; Pelletier et al., 2005; Drakeford et al., 2006; Danion et al., 2007; van Erp et al., 2008; Libby et al., 2013); and (2) numerous animals models for schizophrenia have shown deficits in novel object recognition tasks (e.g., Schneider and Koch, 2003; Brosda et al., 2011; McIntosh et al., 2013).

Regarding early neonatal TTX inactivation of temporal or prefrontal regions, a number of different mechanisms could account for the loss of behavioral LI expression observed in adulthood. It is well accepted that electrical activity plays an essential role in the early development of the nervous system (Spitzer, 2006) and is involved in a number of cellular processes, such as axons’ myelination (Demerens et al., 1996), rearrangement of synaptic connections in target structures (Stryker and Harris, 1986; Katz and Shatz, 1996; Hutchins and Kalil, 2008), and maturation of dendritic spines (Drakew et al., 1999). Importantly, the first 2 postnatal weeks are known to be critical for the development of the parahippocampal regions and prefrontal cortex in rodents (Clancy et al., 2001). Indeed, several authors have reported that the connectivity of the parahippocampal region (i.e., entorhinal cortex and the ventral subiculum), but also of the prefrontal cortex, is still maturing during the second postnatal week (Schlessinger et al., 1975; Singh, 1977a,b; Fricke and Cowan, 1978; van Eden et al., 1990). Thus, following TTX neonatal inactivation, failure of one or more of the abovementioned mechanisms could result in abnormalities in the intrinsic and/or extrinsic connectivity of these brain structures. Through a combination of the aforementioned cellular mechanisms, early TTX inactivation could very well induce a functional disturbance of the entorhinal cortex and ventral subiculum, resulting in encoding defects of the CS but also a loss of recovery of information related to the CS which would manifest itself in adulthood in a malfunctioning recognition memory system, preventing proper learning and memorization of the characteristics related to the CS (banana odor) during pre-exposure to the stimulus (see discussion above; Louilot et al., 2010). Indeed, at adulthood, during the retention session, only the association between the CS and the negative reinforcement (malaise induced by LiCl) that occurred during the conditioning phase would be retrieved. This would explain the expression of an aversive reaction to the stimulus—instead of an approach reaction typical of LI expression—in animals neonatally microinjected with TTX (Peterschmitt et al., 2007; Meyer et al., 2009).

Regarding the prefrontal cortex, the data we have obtained thus far after neonatal inactivation do not allow us to conclude whether or not this structure is involved in encoding the information related to the CS during pre-exposure and/or retrieval during the test phase (Meyer and Louilot, 2012). However, because of close anatomical connections between the prefrontal cortex and the entorhinal cortex and ventral subiculum (Jay et al., 1989; Jay and Witter, 1991; Carr and Sesack, 1996; Insausti et al., 1997; Heidbreder and Groenewegen, 2003; Hoover and Vertes, 2007), the prefrontal cortex may very well be part of the recognition memory system thought to be involved in the LI phenomenon and may thus be malfunctioning after early neonatal inactivation. The disappearance of LI behavior observed in animals subjected to neonatal TTX inactivation may also be related to neurodevelopmental disturbances in target regions of the prefrontal cortex that are secondary to the neonatal inactivation (e.g., myelination defects in projection structures, particularly the hippocampal regions), rather than to a functional impairment of the prefrontal cortex *per se*. Myelination defects have been observed after neonatal ibotenic lesion of the prefrontal cortex (Schneider and Koch, 2005; Klein et al., 2008) but have yet to be demonstrated following TTX postnatal blockade.

Taken together, the data obtained in our early life disconnection model show that neonatal transient blockade of the entorhinal cortex, ventral subiculum, or prefrontal cortex, all structures described as targets of neurodevelopmental disturbances in schizophrenia, disrupt the behavioral expression of LI in adulthood. Based on the experimental and clinical data set out above, it is tempting to propose that these early functional disconnections may induce neurodevelopmental abnormalities in the parahippocampal region and prefrontal cortex which lead in adulthood to a disruption of mnemonic processing abilities resulting in turn in an impaired recognition memory reflected in our animal model by a disruption of the behavioral expression of LI.

## Dysregulation of the striatal LI-related dopaminergic responses

### Involvement of the parahippocampal region: entorhinal cortex and ventral subiculum (see summarizing Figure 2)

We showed very interestingly that the reversal of the behavioral expression of LI following early inactivation of the entorhinal cortex, ventral subiculum or prefrontal cortex had different consequences for LI-related dopaminergic responses depending on the striatal region considered: ventral striatum or dorsal striatum (see summarizing Table 1). As a result of reciprocal connections between the entorhinal cortex and ventral subiculum (Van Groen and Lopes da Silva, 1986; Naber et al., 2000; van Groen et al., 2003; O’Mara, 2005) one would expect the entorhinal cortex and ventral subiculum to exert similar controls on dopaminergic responses at the level of the nucleus accumbens. However, our results showed that a neonatal functional blockade of the entorhinal cortex induced a complete loss of dopaminergic responses characteristic of LI recorded in the core part of the nucleus accumbens, but only a partial reversal of these responses in the dorsal striatum (Peterschmitt et al., 2007; Meyer et al., 2009). Neonatal inactivation of the ventral subiculum, on the other hand, caused partial loss of dopaminergic responses characteristic of LI in the core part and a total disappearance of these responses in the dorsal striatum (Meyer et al., 2009; Meyer and Louilot, 2011). It is clear from these results that the two medial temporal structures exercise a different control over dopaminergic LI-related responses in the ventral and dorsal striatum. Connectivity differences may very well account for the stronger effect of a neonatal functional blockade of the entorhinal cortex on LI-related dopaminergic responses recorded in the core part of the nucleus accumbens. Indeed, there is clear evidence for regulation of the dopaminergic transmission at the level of the nucleus accumbens by the entorhinal cortex, on the one hand (Louilot and Le Moal, 1994; Louilot and Choulli, 1997), and the ventral subiculum, on the other hand (Louilot and Le Moal, 1994; Blaha et al., 1997; Legault et al., 2000; Floresco et al., 2001; Peleg-Raibstein and Feldon, 2006). Secondly, the two parahippocampal regions project on the nucleus accumbens (McGeorge and Faull, 1989), although such projections are distributed unevenly, with a much denser innervation of the core part of the nucleus accumbens from the entorhinal cortex than from the ventral subiculum (Groenewegen et al., 1987; Brog et al., 1993; Totterdell and Meredith, 1997).

As for the dorsal striatum, it seems that, similar to the core part of the nucleus accumbens, the two temporal structures exert a distinct control over the LI-related dopaminergic responses recorded in the anterior part of the dorsal striatum, with a stronger effect induced by inactivation of the ventral subiculum. Although the interactions between the entorhinal cortex and ventral subiculum, with dopaminergic neurons innervating the anterior part of the dorsal striatum, have yet to be specified, known connections mainly originating in the entorhinal cortex and ventral subiculum have been described in the median part of the anterior dorsal striatum, which is where dopamine was measured in our own studies (Groenewegen et al., 1987; McGeorge and Faull, 1989; Finch et al., 1995; Finch, 1996; Totterdell and Meredith, 1997).

**Figure 2:**
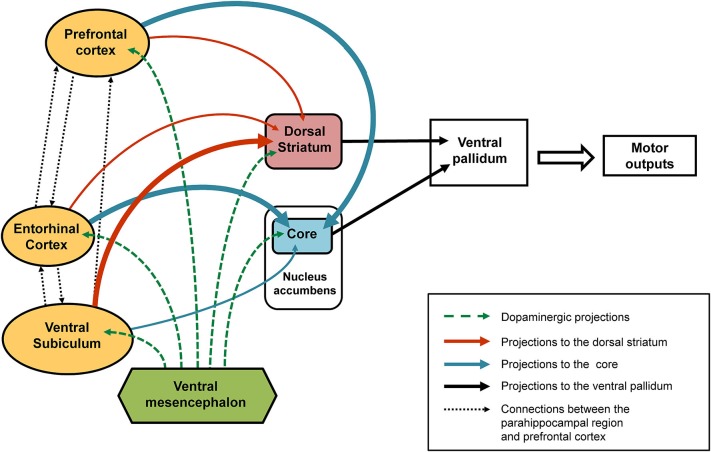
**Schematic representation of the connections affected following early-life (PND8) TTX inactivation.** After TTX blockade of the entorhinal cortex, ventral subiculum or prefrontal cortex performed at PND8 a number of interconnected brain regions are affected (see solid arrows). Our data showed that the ventral subiculum exerts strong control over the dopaminergic responses recorded in the dorsal striatum (thick orange arrows) and only slightly affects the dopaminergic variations in the core part of the nucleus accumbens (thin blue arrow). By contrast, strong control over the dopaminergic responses recorded in the core (thick blue arrows) is exerted by the entorhinal cortex and the medial prefrontal cortex, whereas both these regions have little influence over the dopaminergic responses recorded in the dorsal striatum (thin orange arrows). The broken green arrows denote dopaminergic projections from the ventral mesencephalon. We propose that the behavioral responses observed in latent inhibition are the result of functional complementarity at the level of the ventral pallidum (dorsolateral part), of dopamine-regulated efferents from the dorsal striatum and the core part of the nucleus accumbens

As regards the core part of the nucleus accumbens, a relationship was found in our behavioral paradigm between dopaminergic variations and the direction of behavioral responses (attraction or aversion) in animals not subjected to any neonatal functional blockade (Jeanblanc et al., 2002, 2004; Peterschmitt et al., 2008). Thus, it has been shown that the approach of the stimulus is accompanied by a marked increase in dopamine in the core part of the nucleus accumbens, while a rapid transient signal decrease and return to baseline values accompanies the response of avoidance. Results that support this view have been obtained during self-administration of cocaine (Phillips et al., 2003) in studies where the authors revealed gradual increases in extracellular dopamine levels in rats who were approaching the lever used to deliver cocaine and thus signaling reward, the suggestion being that dopaminergic variations recorded in the core part of the nucleus accumbens may precede the expression of behavioral responses. This interpretation is also consistent with the proposal that the core part of the nucleus accumbens is involved in the permutation or switching between different behavioral sequences so that there is an appropriate response is to the context, and in particular the LI phenomenon (Weiner and Feldon, 1997; Weiner, 2003). Concerning the dorsal striatum, it has also been shown that dopaminergic responses recorded in this region are in the same direction and similar to those found in the core part of the nucleus accumbens (Jeanblanc et al., 2003, 2004; Peterschmitt et al., 2005). However, the dopaminergic responses obtained after neonatal TTX inactivation of the entorhinal cortex and ventral subiculum suggest the relationship between dopaminergic changes in the core and the dorsal striatum and behavioral responses may be more complex than initially suggested. Indeed, pre-exposed conditioned animals that underwent neonatal inactivation of the ventral subiculum surprisingly displayed a small increase in dopamine in the core while displaying an aversion response (Peterschmitt et al., 2007; Meyer and Louilot, 2011). They were also characterized by no increase in dopamine in the anterior part of the dorsal striatum (Meyer et al., 2009). As previously discussed, in pre-exposed conditioned animals subjected to neonatal blockade of the entorhinal cortex an almost opposite dopaminergic profile is obtained in the two striatal regions while a similar behavioral expression is obtained. This important and very interesting finding prompts us to suggest that the lack of dopamine increase in one of the two striatal subregions—core or dorsal striatum—appears to be enough to disrupt the normal behavioral expression of LI. This proposal presupposes a functional similarity and complementarity between the core part of the nucleus accumbens and the adjacent part of the anterior dorsal striatum targeted in our studies. Several reports back the plausibility of this hypothesis. Based on anatomo-functional considerations, Voorn et al. (2004) have proposed that the striatum can be organized in parallel vertical columns encompassing the well-acknowledged ventral and dorsal striatal subdivisions. According to this scheme, the core part of the nucleus accumbens and anterior part of the dorsal striatum situated above the core are part of the same column (Voorn et al., 2004). Moreover, converging efferents from the core part of the nucleus accumbens and adjacent regions of the dorsal striatum close to the dorsolateral part of ventral pallidum have been described (Mogenson et al., 1983; Groenewegen et al., 1993), suggesting the coordinated involvement of the two striatal subregions in output responses, and namely motor responses. It is important to note here that the dorsolateral part of the ventral pallidum has been shown to send direct projections to the substantia nigra, pars reticulata (Groenewegen et al., 1993; see also Zahm, 2000). Our proposal, therefore, is that in LI-related responses there is a functional complementarity between the core and anterior dorsal striatum influences, at the level of the ventral pallidum (see summarizing Figure 2). In this context, it is interesting to note that Menon and colleagues (Menon et al., 2001) used functional magnetic resonance imaging (fMRI) to show a dysfunction of the basal ganglia and more specifically a lack of activation of the putamen and globus pallidus, the suggestion being that these structures are involved in behavioral disturbances in goal-directed actions observed in schizophrenia patients.

### Involvement of the prefrontal cortex (see summarizing Figure 2)

Concerning the neonatal inactivation of the medial prefrontal cortex (infralimbic/prelimbic region), we showed that early and reversible functional inactivation of this region is sufficient to induce a complete loss of behavioral expression of LI as well as a complete disruption of LI-related dopaminergic responses in the core part of the nucleus accumbens (Meyer and Louilot, 2012) and partial loss of these responses in the anterior part of dorsal striatum (unpublished results; see summarizing Table 1). Therefore, these data argue in favor of involvement of the prefrontal cortex in the LI phenomenon, despite its involvement having been controversial for many years (Broersen et al., 1996; Ellenbroek et al., 1996; Joel et al., 1997; Lacroix et al., 1998; Broersen et al., 1999; Lacroix et al., 2000a,b). As mentioned above, the absence of LI in adult animals after postnatal TTX inactivation of the prefrontal cortex could be explained by different cellular mechanisms. Similar to previous studies, no gross anatomical changes were observed in either the prefrontal cortex, nucleus accumbens (Meyer and Louilot, 2012), or dorsal striatum (unpublished results). It is therefore possible to suggest that a transient blockade of the electrical activity in the prefrontal cortex during a critical period involves one or more mechanisms, permanently affecting communication between the prefrontal cortex and structures receiving direct projections such as the nucleus accumbens (Gorelova and Yang, 1997; Heidbreder and Groenewegen, 2003), structures that innervate the prefrontal cortex, such as the subiculum (Jay et al., 1989; Jay and Witter, 1991), or those sharing reciprocal connections with the prefrontal cortex, such as the entorhinal cortex (Insausti et al., 1997; Heidbreder and Groenewegen, 2003; Hoover and Vertes, 2007). As already discussed above, different cellular mechanisms may result in neuroanatomical changes responsible for poor integration of information in the prefrontal cortex resulting in changes in dopamine release in the core part of the nucleus accumbens or the dorsal striatum in pre-exposed conditioned animals subjected to TTX inactivation.

As regards the core part of the nucleus accumbens, it has been shown that dopaminergic transmission depends on the functional activity of the prefrontal cortex (Louilot et al., 1989). Moreover, the prefrontal cortex may control dopaminergic responses directly in the nucleus accumbens. Indeed, several authors have described projections from the infralimbic/prelimbic region of the prefrontal cortex to the core part of the nucleus accumbens (Berendse et al., 1992; Wright and Groenewegen, 1995; Gorelova and Yang, 1997). In addition, a close apposition has been described between efferents of the prefrontal cortex and dopaminergic endings in the nucleus accumbens (Sesack and Pickel, 1992). The prefrontal cortex may also indirectly regulate the activity of dopaminergic neurons reaching the nucleus accumbens via prefrontal efferences in the ventral tegmental area (Carr and Sesack, 2000). Concerning the dorsal striatum, several authors have also reported that striatal dopaminergic transmission is regulated by the prefrontal cortex (Jaskiw et al., 1990; Taber and Fibiger, 1993). It has been shown that there are projections from the infralimbic/prelimbic prefrontal region to the dorsal striatum (Montaron et al., 1996; Heidbreder and Groenewegen, 2003). A close proximity of prefronto-striatal projections and striatal dopaminergic terminals has also been described (Wang and Pickel, 2002). In addition, striatal dopaminergic transmission may be regulated indirectly by prefrontal projections in the substantia nigra (Heidbreder and Groenewegen, 2003). Given the similarities between the prefrontal and ventral and dorsal parts of the striatum in terms of anatomofunctional relationships, it is tempting tentatively to propose that differences in LI-related dopaminergic responses observed in the core part of the nucleus accumbens (complete reversal) and the anterior dorsal striatum (partial reversal) could be related to different functional relationships with another forebrain structure, namely the basolateral nucleus of the amygdala, which plays a crucial role in the control of dopaminergic response characteristics of conditioned aversion in the core part of the nucleus accumbens but does not appear to be involved in these dopaminergic responses in the dorsal striatum (Louilot and Besson, 2000). Importantly, convergence of afferents from the prefrontal cortex and the basolateral nucleus of the amygdala has been described in the nucleus accumbens (Wright and Groenewegen, 1995). Furthermore, amygdala regulation of dopaminergic transmission in the nucleus accumbens appears to be under the influence of the prefrontal cortex (Jackson and Moghaddam, 2001). To the best of our knowledge, such anatomomical and functional relationships have not been reported for the dorsal striatum. Ultimately, it is possible to propose that the disruption of LI-related dopaminergic responses observed following neonatal reversible TTX blockade of the prefrontal cortex could result, in adulthood, from an impairment of the control, mainly glutamatergic, exerted by the prefrontal cortex (Morari et al., 1998; David et al., 2005) over dopamine in the nucleus accumbens, by releasing the amygdalar regulation. However, the precise mechanisms of such disrupted control by the prefrontal cortex in LI following neonatal TTX inactivation are still unclear, given that the prefrontal cortex also seems to regulate the activity of dopaminergic neurons innervating the nucleus accumbens via cortical projections reaching the ventral tegmental area (Carr and Sesack, 2000). Finally, it cannot be excluded that LI-related dopaminergic variations in animals microinjected with TTX in the prefrontal cortex reflect also functional interactions with other forebrain regions, namely the entorhinal cortex and the ventral subiculum.

## Summary and conclusions

Taken together, functional disconnection neurodevelopmental models based on transient neonatal TTX inactivation are an attractive alternative to the NVHL model, since they result in schizophrenia-relevant features without anatomical lesions. Perinatal TTX inactivation consequences on myelination (Demerens et al., 1996), refinement of synaptic connections (Stryker and Harris, 1986; Katz and Shatz, 1996) and normal maturing of dendrites (Drakew et al., 1999; Frotscher et al., 2000) appear to be adequate for modeling disconnections as proposed for schizophrenia (see Section Introduction). Therefore, as regards the heuristic animal models for schizophrenia, postnatal TTX inactivation is an approach that presents a better construct validity. The data collected by our group showed that early functional blockade of the entorhinal cortex, ventral subiculum and prefrontal cortex structures reported in the literature as targets for neurodevelopmental disorders (see Akbarian et al., 1993a,b; Jakob and Beckmann, 1994; Weinberger et al., 1994; Goldman-Rakic and Selemon, 1997; Arnold, 2000; Falkai et al., 2000; Garey, 2010) results in the complete disappearance in adult animals of the LI phenomenon which in several studies has been found to be disrupted in acute patients with schizophrenia (Baruch et al., 1988; Gray et al., 1992, 1995; Lubow et al., 2000; Rascle et al., 2001; Young et al., 2005). Our findings suggest that the functional integrity of each of the three integrative structures is needed for normal expression of LI. We propose that the disappearance of LI behavioral and dopaminergic responses in our paradigm may be due to an impaired treatment of memories concerning the olfactory stimulus occurring at the time of pre-exposure and ultimately causing stimulus recognition in the retention phase and hence proper behavioral expression to be disrupted. Both parahippocampal regions, the entorhinal cortex and subiculum, as well as the medial prefrontal cortex could be part of a distributed system consisting of brain regions that are part of a system involved in stimulus recognition memory which interacts with another system involved in assigning an aversive valence to the stimulus. It is important to note here that a defect of recognition memory with recollection has been reported in patients with schizophrenia (Danion et al., 1999; Huron and Danion, 2002; Pelletier et al., 2005; Drakeford et al., 2006; Danion et al., 2007; van Erp et al., 2008; Libby et al., 2013). It is tempting to propose that neurodevelopmental defects of the same structures (temporal and prefrontal) result in a disruption of mnemonic processing capabilities and loss of LI expression in schizophrenia patients. One question still in abeyance is whether the abnormalities observed at the level of the entorhinal cortex, ventral subiculum or medial prefrontal cortex of patients with schizophrenia correspond to a group of patients or whether these defects can be observed in one and the same patient insofar as it would appear that there are no brain regions that have been found to be consistently affected in patients with schizophrenia (Goldman-Rakic and Selemon, 1997; Gur et al., 2007). Rather, the abnormalities are found in cortical or subcortical regions sharing connections with the prefrontal cortex. It would therefore be tempting to suggest that the expression of schizophrenia is the result of neurodevelopmental defects which occur in one of the cerebral regions that are part of the abovementioned recognition memory system but which engender specific dopamine changes depending on which brain region is affected. This could explain why schizophrenia manifests itself in some many different ways in patients with the disease.

Finally, recently obtained data showed that animals that underwent early-life inactivation of the prefrontal cortex also displayed greater behavioral and neurochemical reactivity to D-amphetamine (Meyer and Louilot, 2012) and ketamine, at subanesthetic doses (Usun et al., 2013). This is of particular interest given that both these drugs are known to induce psychotic symptoms in healthy individuals and to worsen such symptoms in patients with schizophrenia. Taken together, our findings give cause to consider that our conceptual and methodological approach, which integrates the hypothesis of functional disconnections stemming from neurodeveloppemental failures, is valid for modeling the pathophysiology of schizophrenia in animals and, more specifically, an interesting tool for modeling some of the cognitive deficits observed in the disease.

## Conflict of interest statement

The authors declare that the research was conducted in the absence of any commercial or financial relationships that could be construed as a potential conflict of interest.
